# A population-based prospective study on obesity-related non-communicable diseases in northern Iran: rationale, study design, and baseline analysis

**DOI:** 10.3389/fendo.2024.1329380

**Published:** 2024-04-09

**Authors:** Nima Motamed, Farhad Zamani, Mansooreh Maadi, Hossein Ajdarkosh, Farzin Roozafzai, Hossein Keyvani, Hossein Poustchi, Ramin Shakeri, G. Hossein Ashrafi, Dhayaneethie Perumal, Behnam Rabiee, Maziar Moradi-Lakeh, Mahmoodreza Khoonsari, Zahedin Kheyri, Masoud Reza Sohrabi, Azam Doustmohammadian, Bahareh Amirkalali, Fahimeh Safarnezhad Tameshkel, Esmaeel Gholizadeh, Seyed Hamzeh Hosseini, Mohammad Hadi Karbalaie Niya

**Affiliations:** ^1^Department of Social Medicine, Zanjan University of Medical Sciences, Zanjan, Iran; ^2^Gastrointestinal and Liver Diseases Research Center, Iran University of Medical Sciences, Tehran, Iran; ^3^Liver and Pancreatobiliary Diseases Research Center, Digestive Diseases Research Institute, Tehran University of Medical Sciences, Tehran, Iran; ^4^Digestive Oncology Research Center, Digestive Diseases Research Institute, Tehran University of Medical Sciences, Tehran, Iran; ^5^Cancer Theme SEC Faculty, Kingston University, London, United Kingdom; ^6^Faculty of Science, Engineering and Computing, Kingston University, London, United Kingdom; ^7^Montefiore Medical Center, Albert Einstein College of Medicine, Bronx, NY, United States; ^8^Preventive Medicine and Public Health Research Center, Iran University of Medical Sciences, Tehran, Iran; ^9^Department of Internal Medicine, Arak University of Medical Sciences, Arak, Iran; ^10^Psychiatry and Behavioral Sciences Research Center, Addiction Institute, Mazandaran University of Medical Sciences, Sari, Iran

**Keywords:** cardiovascular disease, cohort study, metabolic disorder, obesity, risk assessment

## Abstract

**Background:**

Iran is facing an epidemiological transition with the increasing burden of non-communicable diseases, such as obesity-related disorders and cardiovascular diseases (CVDs). We conducted a population-based prospective study to assess the prevalence and incidence rates of CVDs and obesity-related metabolic disorders and to evaluate the predictive ability of various CVD risk assessment tools in an Iranian population.

**Method:**

We enrolled 5,799 participants in Amol, a city in northern Iran, in 2009–2010 and carried out the first repeated measurement (RM) after seven years (2016–2017). For all participants, demographic, anthropometric, laboratory, hepatobiliary imaging, and electrocardiography data have been collected in the enrollment and the RM. After enrollment, all participants have been and will be followed up annually for 20 years, both actively and passively.

**Results:**

We adopted a multidisciplinary approach to overcome barriers to participation and achieved a 7-year follow-up success rate of 93.0% with an active follow-up of 5,394 participants aged 18–90 years. In the RM, about 64.0% of men and 81.2% of women were obese or overweight. In 2017, about 16.2% and 5.2% of men had moderate or severe non-alcoholic fatty liver disease, while women had a significantly higher prevalence of metabolic syndrome (35.9%), and type 2 diabetes mellitus (20.9%) than men. Of 160 deceased participants, 69 cases (43.1%) died due to CVDs over seven years.

**Conclusion:**

The most prevalent obesity-related chronic disease in the study was metabolic syndrome. Across the enrollment and RM phases, women exhibited a higher prevalence of obesity-related metabolic disorders. Focusing on obesity-related metabolic disorders in a population not represented previously and a multidisciplinary approach for enrolling and following up were the strengths of this study. The study outcomes offer an evidence base for future research and inform policies regarding non-communicable diseases in northern Iran.

## Introduction

The global prevalence of chronic non-communicable diseases (NCDs) is rising (1, 2). NCDs are the primary cause of morbidity and mortality (1, 2). According to the World Health Organization, by 2030, NCDs will account for 80% of mortalities worldwide (2). Iran, a low- and middle-income country (LMIC), is facing an epidemiological transition with the increasing burden of NCDs (3). According to a systematic analysis for the global burden study 2019, NCDs were responsible for 15.5 million (78.1%) disability-adjusted life years (DALYs) in Iran, with a 35% increase compared with 1990 (44.2%) (4).

Trends in obesity and obesity-related metabolic disorders, such as non-alcoholic fatty liver disease (NAFLD), type 2 diabetes mellitus (T2DM), and metabolic syndrome (MetS), are rising worldwide, and particularly in Iran, due to lifestyle behaviors, such as physical inactivity, and high-calorie diets (5). Studies showed a critically increased prevalence of obesity in adults and children during previous decades, such that 2.1 billion individuals were identified as overweight or obese (6). Exacerbation of anthropometric characteristics of a population might contribute to an increase in mortality and morbidity rates in that population. Studies observed that for every 5-unit increase in the body mass index (BMI) above 25 kg/m^2^, the mortality rate increased by 29% (7, 8). The increasing prevalence of obesity and T2DM is associated with the rising trend of NAFLD, an emerging healthcare challenge in Asia that is prevalent in about a third of Iranian adults (9–12).

Meanwhile, cardiovascular diseases (CVDs) and their associated risk factors are major burdens for global health (13, 14). Causing 46% of global mortality, CVDs are the leading cause of death worldwide (2, 13, 14). Approximately 42% of NCD-related premature deaths are attributed to CVDs (2). Following the global trends, Iran is experiencing high burdens of CVDs, accounting for 51% of deaths in the country (2, 15–17). Unlike LMICs, high-income countries have decreased CVD-related mortality rates through effective preventive approaches and robust therapeutic interventions in recent years (18).

Although a close relationship was ascertained between metabolic disorders, CVDs, and NAFLD (19–22), large community-based longitudinal studies can provide comprehensive data for assessing demographic, epidemiological, and molecular determinants of these major burdens of diseases (3, 23). This, in turn, can affect the efficiency of public health systems, the development, and implementation of community-tailored predictive tools, informing of policy, and the adoption of new public health initiatives for improving socioeconomic determinants of health and alleviating their impact on NCD mortality and morbidity (3, 23).

Therefore, we designed and launched a population-based prospective longitudinal study, named Amol Cohort Study (AmolCS), in 2009 with the primary aim of determining the incidence rates and risk factors of NAFLD, metabolic diseases, and CVDs in northern Iran and evaluating the predictive validity of CVD risk assessment tools. We also aimed to develop and validate population-customized risk assessment tools based on the data of an LMIC.

## Methods

### Ethical considerations

**Figure 1:**
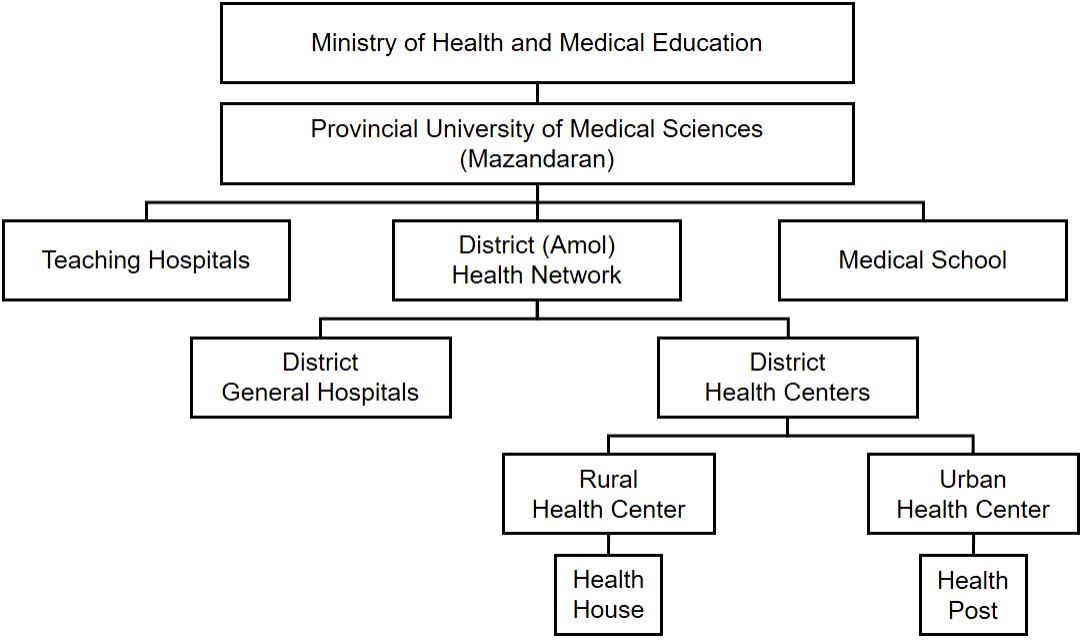
The structure of healthcare network in Iran.

Following the Declaration of Helsinki principles and the STROBE guideline, the study has been designed and conducted with the formal approval of the Research Ethics Committee of Iran University of Medical Sciences (reference number: IR.IUMS.REC.1397.162). All participants freely gave written informed consent prior to participation. We held meetings with the head of the district health network (**Figure 1**) and stated the objectives of the study to gain their cooperation.

### Cohort catchment area

**Figure 2:**
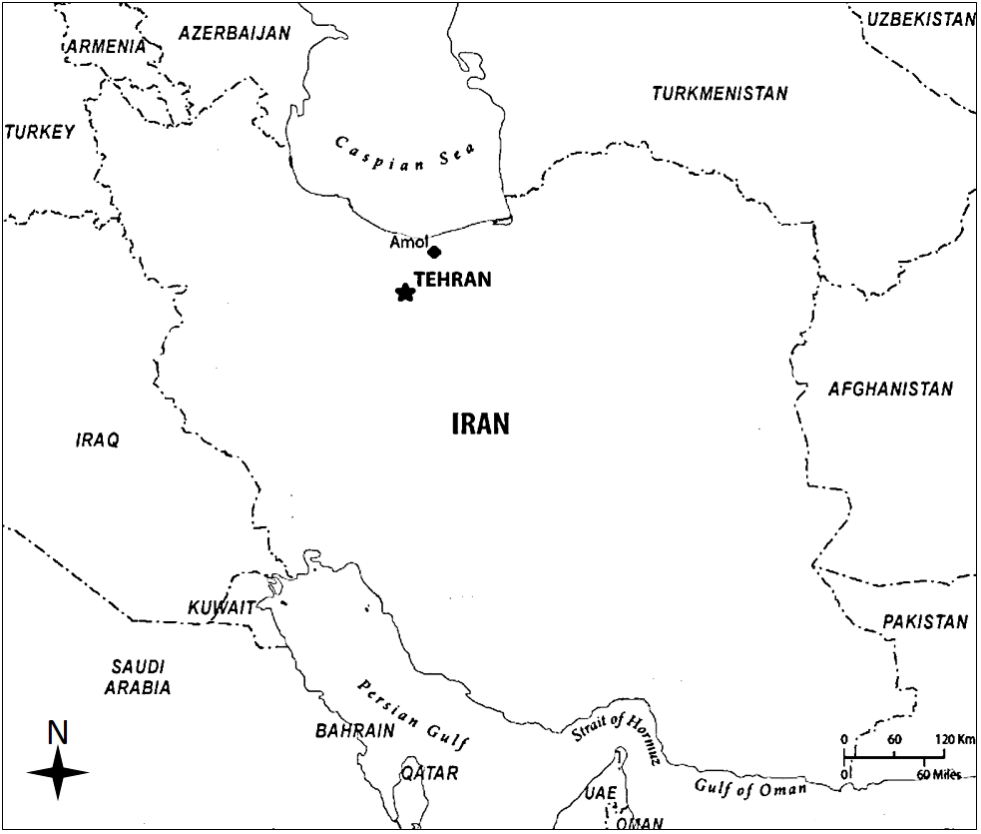
Geographic location of Amol city, the catchment area of the Amol Cohort Study, in northern Iran.

The AmolCS has been conducted in the Haraz research center, covering the general adult population of Amol and its rural surroundings since 2009. This research center is in the Hefdah-e-Shahrivar Hospital, a non-academic district hospital in Amol City. Amol is one of the most populated cities of Mazandaran, a Caspian Sea coastal province in northern Iran (**Figure 2**). The city has a Mediterranean climate with relatively hot summers and cool, humid winters. The main agricultural export product and main food of the population of Amol is high-quality rice. Based on the last national census, the Amol district has 401,639 residents (202,502 men and 199,137 women) in 133,034 households. Of this population, 246,355 (61.3%, 123,158 men and 123,197 women) live in 81,433 urban households, and 155,284 (38.7%, 79,344 men and 75,940 women) live in 51,601 rural households (24). According to the census, Mazandaran province has the highest literacy rate (97.6%) in Iran (24).

### Sample size calculation

Assuming that the expected annual incidence of NAFLD is 10% (based on a previous study) (25–27), and assuming the margin of error of 1% with 95% confidence intervals, and an expected drop-out rate of 50% (to ensure a sufficient sample size to conduct the first cohort study in the region), the following formula estimated that a total of nearly 7,000 people should be recruited (of 339122 target population (24)):

$N=Z_{1-\alpha /2}^{2}\left[p\left(1-p\right)\right]/\delta^{2}$ ;


α=0.05,p=0.1, δ=0.01

Where N: calculated sample size; $Z_{1-\alpha /2}=1.96\;$ based on Z-tables with α=0.05 ; P: expected proportion (annual incidence of NAFLD) based on previous studies (25, 26); α: type one of error; δ: Precision or Margin of Error (assuming 0.1 of expected proportion).

### Multidisciplinary approach for enrollment

Following a multidisciplinary approach through the ministerial hierarchy (**Figure 1**), we cooperated with rural and urban health centers and their respectively affiliated health houses and health posts to recruit and enroll the sample population. Each rural health center supervises several health houses where Behvarzes work. A Behvarz is an employee certified to provide free universal primary healthcare packages, such as vaccination, screening, perinatal care, and environmental health services (e.g., water sanitation), for all registered inhabitants. Behvarzes are selected from indigenous candidates who merit the educational, practical, and ethical prerequisites. They are acquainted with local culture and language and can easily communicate with the locals. We held meetings with Behvarzes, explained the study objectives, and provided a brief tutorial on inviting eligible individuals to the research center. We continuously communicated with Behvarzes to check the progress and update participants’ information. In urban areas, we faced fewer restrictions because the research center was located in the city; thus, accessing the research center and the eligible participants was relatively easier. No expenses were incurred to help with transportation from the participant’s home to the research center. However, we were in continuous contact with the staff of health posts to invite the residents to the research center.

**Figure 3:**
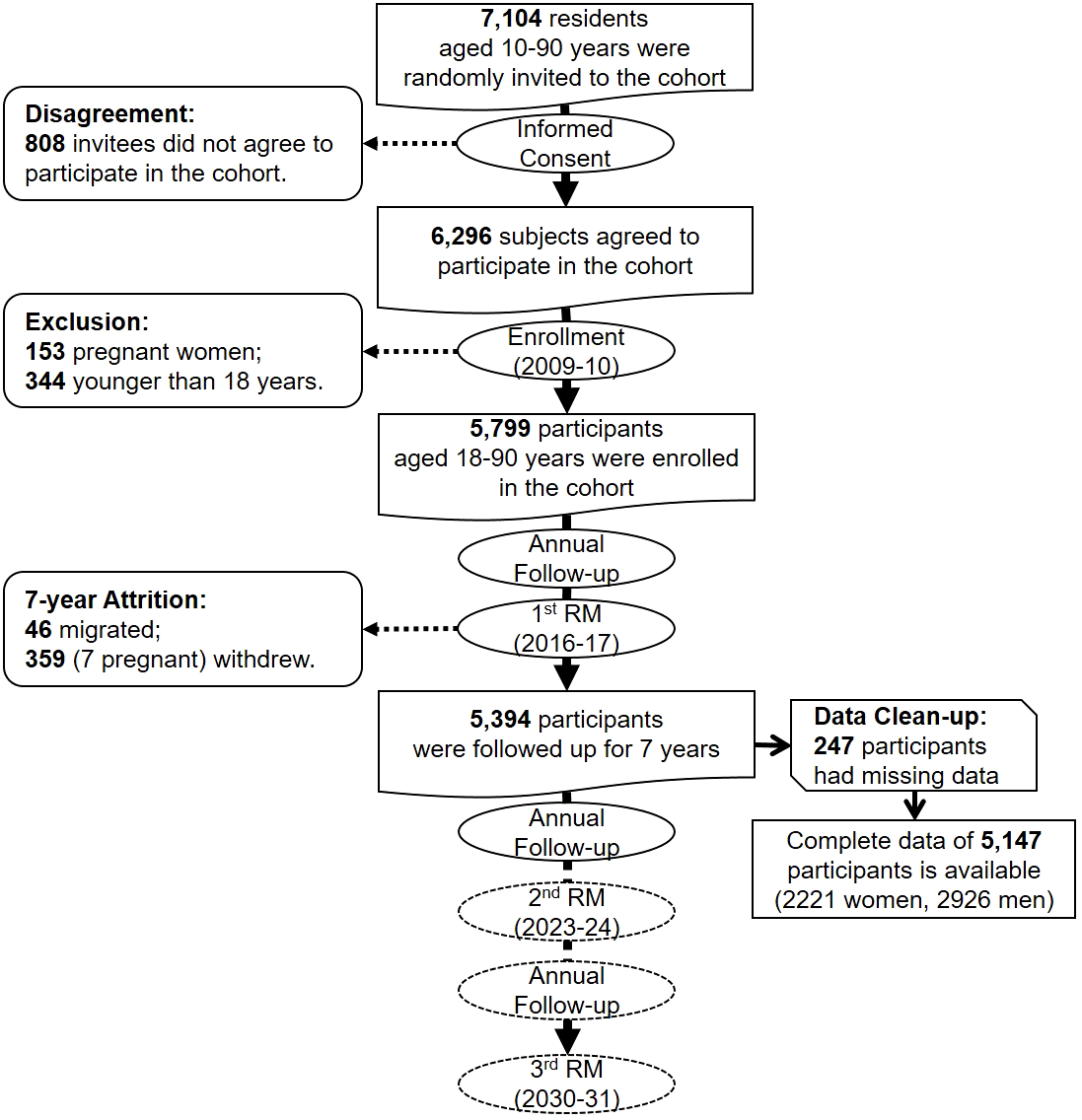
Schematic flowchart of the Amol Cohort Study (RM, repeated measurement).

The primary data (particularly demographics) of residents in rural and urban areas are documented in household records kept in related health houses and posts. Therefore, we developed a sampling frame based on data from health houses and health posts in rural and urban areas. Individuals 10–90 years old were considered eligible for participation in the study. We divided the population into 16 strata based on sex and age groups (10–19, 20–29, 30–39, 40–49, 50–59, 60–69, 70–79, and 80–90 years). Participants were randomly selected from each stratum according to its proportional size. We invited 7,104 individuals, and 88.6% agreed to participate in the cohort. Excluding children (younger than 18 years), sojourners, unwilling individuals, and pregnant women, we enrolled 5,799 eligible residents from the general population of Amol (**Figure 3**).

### Enrollment phase

The enrollment phase was conducted over approximately 18 months (2009–2010). Eighteen personnel with academic degrees in nursing and nutrition conducted interviews, measurements, sample collection and preparation, and data entry. These personnel, who were familiar with both Persian and local languages, were theoretically and practically trained during a 2-week course on the study objectives and protocols.

The enrollees arriving at the research center were informed of the purpose and workflow of the study. We obtained written informed consent (in Persian), stating invasive procedures, the confidentiality of disclosing information, and the unconditional right to withdraw from those willing to participate at any time. Upon registration, a unique code was assigned to each participant to make the data and samples anonymized.

### Blood sampling

A day before the registration, the participants were asked to fast for 12 hours before attending the research center the next morning. Before taking blood, all tubes were labeled appropriately. After registration and following standard protocols, 20 mL of fasting antecubital venous blood sample was obtained from each participant. Half of the blood sample was transferred to a serum separator tube and kept at room temperature for 30 minutes to allow for clotting. These samples were immediately centrifuged at 3000 revolutions per minute (RPM) for 10 minutes. Then, the serum was separated using a clean pipette technique. An appropriate amount of serum was used for biochemical assessments, and the remaining serum was aliquoted into labeled 500 μL cryotubes (Eppendorf Intl., Hamburg, Germany) and stored at -80°C. The other half of the blood sample was used for plasma separation from an ethylene-diamine-tetraacetic acid-containing collection tube and centrifuged at 3000 RPM for 10 minutes. After centrifugation using a clean pipette technique, 1.0 mL of plasma was separated, aliquoted into labeled 1.5 mL cryotubes, and stored at -80°C.

### Anthropometry

Anthropometric indices were measured after sample collection while the participant was still fasting. The participant was then provided with a snack. Weight (in kg) was measured using a digital scale (Seca GmbH & Co.KG, Hamburg, Germany) after the participant removed her/his shoes and excess clothes. It was recorded as rounded to the nearest 0.1 kg. Also, the height (rounded to the nearest 0.5 cm) of barefoot participants was measured in an upright position with their heels, buttocks, and scapulae pressed up against a wall, using a wall-mounted manual stadiometer (Seca GmbH & Co.KG, Hamburg, Germany). The participant’s weight was divided by the square of the height (kg/m^2^) to calculate the BMI.

Waist circumference (rounded to the nearest 0.1 cm) was measured using a plastic tape measure in a horizontal plane at the midpoint between the lowest costal ridge and the upper border of the iliac crest, in standing posture and at the end of a gentle expiration. While standing with the legs close together, the largest circumference between the waist and knee was measured using the tape, rounded to the nearest 0.1 cm, and recorded as the participant’s hip circumference (in cm). Waist circumference was divided by hip circumference to calculate the waist-to-hip ratio.

### Dietary assessments

Dietary assessment was conducted using dietary habits and a validated semi-quantitative food-frequency questionnaire (FFQ) which was completed during face-to-face personal interviews by expert dietitians (28). The dietary habit questionnaire was completed during the first interview to obtain the usual dietary habits. It included questions about the consumption of fried and fast foods, fruits and vegetables, snacks, meats, and dairy products. Dietary data related to past years were collected using the 168-item semi-quantitative FFQ adopted from the Willet format (29). Participants were asked to estimate their consumption of different food items on a daily, weekly, or monthly basis. The reported portion size and frequency of food intake are converted to daily intake, and the portion size is converted to grams by household measures (30). FFQ questionnaire was completed in the first RM.

### Questionnaires

All questionnaires (in Persian) were checked for completeness by the data supervisor. Data on sex, age, marital status, educational level, occupation, and residency status (urban and rural) were collected upon registration. The interviewers asked about the medical history of the participants and their family members (not reported), particularly for CVDs (hypertension, ischemic heart disease, heart failure, cerebrovascular accidents (CVA), and other CVDs), T2DM, dyslipidemia, asthma, obstetrics history, gynecological disorders, malignancies, hospitalization, surgical procedures, chemotherapy or radiotherapy, transfusion, and cause-specific death. They also investigated the participants’ medication history, particularly anti-hypertensive, anti-coagulant, anti-hyperglycemic, anti-lipemic, and anti-asthmatic medications.

In the enrollment phase, personal habits (tobacco smoking, alcohol consumption, and narcotics abuse) were assessed qualitatively (Yes/No). The severity and duration of physical activity were evaluated using the validated Persian translation of the international physical activity questionnaire (31). Interviewers also completed a 12-item general health questionnaire and a 12-item short-form health survey questionnaire (in Persian) (32). In addition, a validated Persian gastroesophageal reflux disease questionnaire was completed for each participant (33).

### Clinical evaluations

Blood pressure (in mmHg) was measured twice using a fitted sphygmomanometer cuff (Riester GmbH, Jungingen, Germany) while the participant was in a sitting position after at least five minutes of inactivity in a quiet room. Systolic and diastolic blood pressures were determined based on Korotkoff sounds. The average of two measured values was considered as the participant’s blood pressure.

A single expert radiologist performed hepatobiliary ultrasonography using a 3–5 MHz transducer (Esaote SpA, Genova, Italy). Sagittal, longitudinal, lateral, and intercostal views of the liver parenchyma and biliary system were obtained, and all findings, including hepatomegaly, steatosis, cirrhosis, cholelithiasis, cysts, polyps, calcifications, hemangioma, and space-occupying lesions were recorded.

### Laboratory assessments

We enzymatically assessed serum samples using an Auto-Analyzer BS200 (Mindray, Shenzhen, China) and diagnostic kits (Pars Azmoon Co., Tehran, Iran) for fasting blood sugar, insulin, total cholesterol, high-density lipoprotein cholesterol, low-density lipoprotein cholesterol, triglycerides, alanine aminotransferase, aspartate aminotransferase, alkaline phosphatase, and γ-glutamyl transpeptidase. Enzyme-linked immunosorbent assay (ELISA) kits (Pishtaz Teb Co., Tehran, Iran) were used for evaluating hepatitis B surface antigen, hepatitis B surface antibody, hepatitis B core antibody, and hepatitis C virus antibody in the enrollment phase. Then, 10 percent of the blood samples were re-evaluated by the Iranian National Reference Laboratory. The coefficients of variation ranged from 1.7% to 3.8% for all laboratory values. All test results were recorded, and a copy was provided to the participant.

### Outcomes of interest

The outcomes of interest in the AmolCS include the number of cause-specific deaths, the incidence of CVDs, metabolic disorders, including MetS and T2DM; liver diseases, including NAFLD, and viral hepatitis B and C; and trends in major risk factors associated with NCDs, including anthropometric measurements, physical activity, and physiological, lifestyle, nutritional, and environmental factors.

### Definitions

1. We included the fatal and non-fatal CVD events in the present study as cardiovascular outcomes. The outcomes of patients were determined based on the report of the patients or a closed family of patients matched with medical records. The related outcomes were considered a history of hospitalization due to myocardial infarction, angiographically proven coronary heart diseases, PCI history, and cerebrovascular diseases.
2. DM type 2 was defined by a history of DM or taking anti-hyperglycemic drugs (oral agents, insulin) or an FBS≥125 mg/dl with no history of DM type I.
3. NAFLD was defined by ultrasonographic evidence of hepatic fat accumulation, i.e., markedly increased hepatic echogenicity, and ruling out other causes of hepatitis as well as secondary steatosis due to alcohol, steatogenic medications, or genetic diseases. Participants were categorized into four groups (Grade 0: normal liver echogenicity; Grade 1: fatty liver with increased liver echogenicity; Grade 2: fatty liver with the echogenic liver obscuring the echogenic walls of the portal venous branches; Grade 3: fatty liver in which the diaphragmatic outline is obscured) (34).
4. Based on the AHA/NHLBI update of ATP III definition (35, 36), MetS diagnostic criteria included any three of the following clinical conditions:

- Triglyceride ≥150 mg/dl or on drug treatment for elevated TG
- Waist circumference ≥102 cm in men and ≥88 cm in women
- Fasting glucose ≥100 mg/dl or on drug treatment for elevated
- Systolic blood pressure ≥130 mm Hg and/or diastolic blood pressure ≥85 mm Hg) or on drug treatment for elevated blood pressure
- HDL-c<40 mg/dl in men and<50 mg/dl in women or on drug treatment for low HDL

### Follow-up phase

After the enrollment, the participants have been and will be actively followed up annually by phone for 20 years. Interviewers were asked about any clinic visits, medical events, and diagnostic and therapeutic procedures a year before the call. In the case of not receiving an answer from a participant following three phone calls, the research team contacted the related health house or health post staff to establish reasons, if any (withdrawal, emigration, or death). To minimize cohort attrition and participant withdrawal, the team offered reassurances and more study details whenever participants required. If the participant was still in the catchment area, i.e., lived in the same postal address given in the enrollment phase or moved to another covered address, the team would contact and invite the participant to a face-to-face meeting to alleviate possible concerns and provide needed information.

During active follow-ups, questionnaires [including a validated Persian verbal autopsy (37)] were and will be completed regarding the occurrence of death and incidence of medical events, hospitalizations, or diagnostic and therapeutic procedures, particularly those related to the outcomes of interest, such as angiography, coronary angioplasty, or cardiac surgery. In case of death or incidence of the outcomes of interest, the participant or her/his family members were asked to bring all relevant medical documents, such as hospital records, and death certificate, to the research center. If participants or their family members could not come to the center, the team would refer to the hospital or the participant’s residence to collect and review copies of pertinent documents and record the data. A trained internist reviewed the medical documents and confirmed the diagnosis of the disease and the date and cause of death.

Passive follow-up was also conducted through self-reports. At the time of enrollment, the participants and their family members were provided with a contact number to inform the research team in the event of death, hospitalization, or the development of major medical events as soon as possible. After receiving self-reports, the team referred to hospitals or participants’ residences, collected and reviewed the medical documents, confirmed the outcomes, and recorded the data. Detailed information on the data collection is presented in **Table 1**.

**Table 1:** Measurements at baseline and follow-up assessments.

Classification	Components	Measures and instruments
Baseline data		
General	Socio-demographic	age, sex, ethnicity, region of residency (rural, urban), educational level, marital status, occupation
	Lifestyle	past/current smoking, alcohol consumption, opium use, hookah smoking
Medical	Medical history and general health	complete personal medical history, family medical history (hypertension, hyperlipidemia, diabetes, CVDs), current use of medicine, quality of life (SF-12 and GHQ-12 questionnaires), gastroesophageal reflux disease questionnaire
	Laboratory assessments	FBS, lipid profile assessments (triglyceride, LDL cholesterol, HDL cholesterol, cholesterol); liver function tests (alanine transaminase [ALT], aspartate transaminase [AST], gamma-glutamyl transaminase [GGT]), serum insulin, hepatitis B virus surface antigen [HBsAg], hepatitis B virus surface antibody [HBsAb], hepatitis B core antibody [HBcAb], hepatitis C virus antibody [HCvAb]
	Anthropometric measurements	weight, height, waist circumference, hip circumference
	Clinical evaluations	NAFLD diagnosis by ultrasound, blood pressure measurement, and standard 12-lead electrocardiogram (ECG) to diagnose cardiac abnormalities
Nutrition	Dietary habits	dietary habits during the past year and current
Follow up data		
General	Socio-demographic	age, sex, ethnicity, region of residency (rural, urban), marital status, educational level, occupation
	Lifestyle	past/current smoking, alcohol drinking (quantitative assessment), opium use, hookah smoking, physical activity (international physical activity questionnaire-IPAQ)
Medical	Medical history and general health	Complete personal medical history, family medical history (hypertension, hyperlipidemia, diabetes, CVDs), current use of medicine, quality of life (SF-12 and GHQ-12 questionnaires), gastroesophageal reflux disease questionnaire, Holmes-Rahe stress questionnaire
	Laboratory assessments	FBS, lipid profile assessments (triglyceride, LDL cholesterol, HDL cholesterol, cholesterol); liver function tests (alanine transaminase [ALT], aspartate transaminase [AST], gamma-glutamyl transaminase [GGT]), C-reactive protein [CRP], glycated hemoglobin A1c [HBA1c], blood urine nitrogen [BUN], creatinine, Ca, P, vitamin D, T3, T4, TSH, microalbuminuria, Anti tTG/IgG, DGP/IgG, hepatitis B virus surface antigen [HBsAg], hepatitis B virus surface antibody [HBsAb], hepatitis B core antibody [HBcAb], hepatitis C virus antibody [HCvAb]
	Anthropometric measurements	weight, height, waist circumference, hip circumference, arm circumferences, wrist circumferences, and neck circumferences
	Clinical evaluations	abdominal ultrasound, blood pressure measurement, and standard 12-lead electrocardiogram (ECG) to diagnose cardiac abnormalities
	Death records	date of death, cause of death (validated autopsy-VA)
Nutrition	Dietary intake	food frequency questionnaire (168 items-in past year)

### Endpoints

The endpoints of the study are death, withdrawal, and migration of participants.

### Repeated measurements

After seven years, we resumed meetings with the head of the district health network to gain their cooperation in the first RM phase (2016–2017). Again, Behvarzes and the health post staff assisted in actively inviting the participants to the research center. The research team communicated with the participants regarding the main goals of the RM phase and invited them by phone call to refer to the research center. Currently, two more RM phases with 7-year intervals are considered for a comprehensive evaluation of participants, commencing in 2023 and 2030.

In the first RM, data on sex, age, marital status, educational level, and residency status were collected again. The interviewers re-investigated the medical history, drug history, family history, and personal habits of the participants. Unlike the enrollment phase, tobacco smoking and alcohol consumption were assessed quantitatively in the first RM. The same questionnaires on physical activity and quality of life were completed again in the first RM. FFQ and the Holmes-Rahe stress questionnaire were also added in the first RM.

Protocols for measurement of weight, height, and circumferences of waist and hip were the same as in the enrollment phase. In addition, arm, wrist, and neck circumferences were measured in the first RM. Arm circumference (rounded to the nearest 0.1 cm) was measured using a plastic tape measure around the anatomically positioned non-dominant upper arm at the midpoint between the acromion and the olecranon and perpendicular to the longitudinal axis of the arm. Wrist circumference (rounded to the nearest 0.1 cm) was measured using the tape positioned over the radial and ulnar styloid processes of the participant’s dominant hand. Neck circumference (rounded to the nearest 0.1 cm) was measured midway through the neck, between the mid-cervical spine and mid-anterior neck, using the plastic tape while the participant was standing and looking straight ahead with shoulders down but not hunched. Trapezius muscles were not involved in the measurement. In the case of a laryngeal prominence, it was measured just below the thyroid cartilage.

Protocols for blood pressure measurement and hepatobiliary ultrasonography were the same as in the enrollment phase. A standard 12-lead electrocardiogram (ECG) was also provided for each participant in the first RM. Duration of the PR interval, amplitude, and duration of QRS complex, ST segment deviations, and duration and abnormalities of the T wave, if any, were documented. In addition to an automated ECG interpretation, two internists evaluated every ECG. All suspected or abnormal cases were referred to and confirmed by an expert cardiologist.

The blood samples were collected and prepared following the same protocols of the enrollment phase. In addition to the same biochemical profiles assessed in the enrollment, we worked with the Auto-Analyzer and diagnostic kits to evaluate serum concentrations of HbA1C, uric acid, BUN, creatinine, CRP, TSH, T3, T4, vitamin D, calcium, and phosphorus in the first RM. Tissue transglutaminase antibody was assessed with ELISA kits in the first RM. We also used an automated blood cell counter (Sysmex K1000, Hamburg, Germany) for complete and differential blood counts, such as hemoglobin concentration, red blood cell count, volume, and morphology. All test results were recorded, and as incentives, the results of clinical and laboratory assessments were provided to all participants. Those with abnormal findings received appropriate counseling and follow-up care if needed.

### Statistical analysis

The baseline characteristics of participants in the enrollment (2010) and first repeated measurement (2017) phases of the study were summarised as numbers with percentages and mean ± SD for categorical and continuous variables, respectively. The χ^2^ test for categorical variables and the t-test for continuous variables were used to determine differences in baseline characteristics and the prevalence of health disorders according to sex. All data analyses were conducted using SPSS version 24 (Statistical Package for Social Science, SPSS Inc, Chicago, IL, USA) software. A 2-tailed p<0.05 was considered statistically significant.

## Results

### Cohort population

We collected the baseline data of 5,799 participants in enrollment and 7-year data of 5,394 participants in the first RM. The data from 247 participants were not appropriate for analysis and were cleaned up. We finally analyzed the data of 5,147 participants (**Figure 3**). In both enrollment and the first RM, men comprised 56.8% of the population. The mean ( ± SD) age was 44.60 ± 16.73 in men and 43.25 ± 15.25 in women. Significant differences were found in hypertension and lipid profile categories between men and women in both enrollment and first RM phases (all p<0.001). Mean BMI, total cholesterol, LDL-C, HDL-C, and FBS were significantly higher in women than men in both phases (all p<0.001). In contrast, systolic and diastolic blood pressures were significantly higher in men than women (p<0.05) (**Table 2**).

**Table 2:** Some basic characteristics of participants in the enrollment (2010) and first repeated measurement (2017) phases of the Amol Cohort Study.

Variable	Categories	Enrollment ^a^		χ ^2^/T ^b^	P-value	First Repeated Measurement ^a^		χ ^2^/T ^b^	P-value
		Men (n = 3294, 56.8%)	Women (n = 2505, 43.1%)			Men (n = 2926, 56.8%)	Women (n = 2221, 43.1%)		
BMI ^c^	Underweight	72 (2.2)	43(1.7)	391.45	<0.001	39(1.3)	33(1.5)	373.16	<0.001
	Normal	1117(33.9)	436(17.4)			1013(34.6)	384(17.3)		
	Overweight	1341(40.7)	794(31.7)			1256(42.9)	786(35.4)		
	Obesity	764 (23.2)	1232(49.2)			618(21.1)	1018(45.8)		
	Mean ± SD	26.5 ± 4.6	29.7 ± 5.7	-21.83	<0.001	26.7 ± 4.5	29.8 ± 5.7	-20.38	<0.001
Blood pressure ^d^	Optimal	1525 (46.3)	1280 (51.1)	21.56	0.001	1485(50.8)	1252(56.4)	28.36	<0.001
	Normal	709 (21.5)	457(18.2)			687 (23.5)	405 (18.2)		
	High normal	413(12.6)	277 (11.1)			299 (10.2)	193 (8.7)		
	Grade 1hypertension	462(14.0)	316(12.6)			321 (11.0)	253(11.4)		
	Grade2 hypertension	150 (4.5)	136 (5.4)			104 (3.5)	86 (3.9)		
	Grade 3hypertension	35 (1.1)	39 (1.6)			30 (1.0)	32 (1.4)		
DBP	Mean ± SD	76.59 ± 12.61	75.76 ± 13.20	2.29	0.022	72.69 ± 11.67	71.21 ± 12.50	4.27	<0.001
SBP	Mean ± SD	117.25 ± 15.55	115.27 ± 17.70	4.25	<0.001	116.60 ± 18.55	114.78 ± 21.20	3.23	0.002
Total Cholesterol ^e^	Desirable	2264(68.7)	1501(59.9)	58.65	<0.001	2156(73.7)	1574(70.9)	21.42	<0.001
	Borderline high	788(23.9)	705(28.2)			612(20.9)	478(21.5)		
	High	222(6.7)	261(10.4)			146(5.0)	142(6.4)		
	Very high	20(0.6)	38(1.5)			12 (0.4)	27(1.2)		
	Mean ± SD	178.6 ± 41.8	188.7 ± 43.0	-8.43	<0.001	176.8 ± 38.1	182.3 ± 40.5	-4.89	<0.001
LDL-C ^f^	Very low	343(10.4)	203(8.1)	23.53	<0.001	380(13.0)	255(11.5)	5.77	0.341
	Low	1074(32.6)	727(29.0)			1259(43.0)	953(42.9)		
	Borderline low	1160(35.2)	908(36.2)			980(33.5)	742(33.4)		
	Borderline high	557(16.9)	512(20.4)			272(9.3)	231(10.4)		
	High	128(3.9)	125(5.0)			32(1.1)	33(1.5)		
	Very high	26(0.8)	30(1.2)			3(0.1)	7(0.3)		
	Mean ± SD	104.9 ± 30.9	109.5 ± 31.4	-5.07	<0.001	94.7 ± 25.4	99.0 ± 26.1	-2.16	0.031
HDL-C ^g^	Low	1512(45.9)	1631(65.1)	174.95	<0.001	1352(46.2)	1488 (67.0)	221.01	<0.001
	Normal	1782(54.1)	874(34.9)			1574 (53.8)	733 (33.0)		
	Mean ± SD	43.5 ± 11.6	46.4 ± 12.1	-8.47	<0.001	41.6 ± 10.8	45.9 ± 11.3	-13.54	<0.001
Triglycerides ^h^	Normal	2174(66.0)	1698(67.8)	0.309	0.152	2089(71.4)	1655(74.5)	4.24	0.39
	High	1120(34.0)	807(32.2)			837 (28.6)	566(25.5)		
	Mean ± SD	144.80 ± 91.86	141.47 ± 98.44	1.11	0.269	134.60 ± 87.27	127.07 ± 82.29	3.10	0.002
NAFLD ^i^	Negative	1937(58.8)	1403(56.0)	4.40	0.037	1586(54.2)	1153(51.9)	23.6	<0.001
	Grade 1	1008(30.6)	774(30.9)			714(24.4)	655(29.5)		
	Grade 2	300(9.1)	278(11.1)			474(16.2)	346(15.6)		
	Grade 3	49(1.5)	50(2.0)			152(5.2)	67 (3.0)		
T2DM ^j^	Negative	2994(90.9)	2094(83.6)	64.65	<0.001	2598(88.8)	1757 (79.1)	80.39	<0.001
	Positive	300(9.1)	411(16.4)			328(11.2)	464 (20.9)		
	FBS: Mean ± SD	98.5 ± 29.9	103.8 ± 41.3	-5.30	<0.001	103.7 ± 31.6	109.3 ± 42.2	-5.35	<0.001
MetS: based on JIS definition	Negative	2243 (68.1)	1333(53.2)	105.79	<0.001	1957 (66.9)	1424 (64.1)	4.19	0.041
	Positive	1051 (31.9)	1172 (46.8)			969 (33.1)	797 (35.9)		

BMI, body mass index; CI, confidence interval; DBP, diastolic blood pressure; HDL-C, high-density lipoprotein cholesterol; JIS, joint interim statement; LDL-C, low-density lipoprotein cholesterol; MetS, metabolic syndrome; n, count; SBP, systolic blood pressure; T2DM, type 2 diabetes mellitus.

a Data are presented as mean (SD) or number (percentage) except for TG is presented as Geometric mean (GM) and the 95% confidence interval is (CI).

b The independent t-test was applied to compare the continuous variables between two sexes (the related Ts and P-values were reported) and the chi-square test was applied to evaluate the association between sex and categorical variables (the related values of χ ^2^ and associated P-values were reported).

c Underweight:<18.5 kg/m^2^, Normal: 18.5–24.9 kg/m^2^, Overweight: 25–29.9 kg/m^2^, Obesity: ≥30 kg/m^2^.

d Optimal: SBP<120 and DBP<80, Normal: SBP 120-129 and/or DBP 80-84, High normal: SBP 130-139 and/or DBP 85-89, Grad 1 of Hypertension SBP 140-159 and/or DBP 90-99, Grad 2 of Hypertension SBP 160-179 and/or DBP 100-109, Grad 3 of Hypertension SBP ≥ 180 and/or DBP ≥110 (38).

e Desirable:<200 mg/dl, Borderline high: 200–239 mg/dl, High: 240–299 mg/dl, Very high: ≥300 mg/dl.

f Very low:<70 mg/dl, Low: 70–99 mg/dl, Borderline low: 100–129 mg/dl, Borderline high: 130–159 mg/dl, High: 160–189 mg/dl, Very high: ≥190 mg/dl.

g Normal: ≥40 mg/dl in men and ≥50 mg/dl in women, Low:<40 mg/dl in men and<50 mg/dl in women.

h Normal:<150 mg/dl, High: ≥150 mg/dl.

i Grades of fatty liver on Ultrasound were defined as Negative: normal liver echogenicity; (Grade 1: fatty liver with increased liver echogenicity; Grade 2 fatty liver with the echogenic liver obscuring the echogenic walls of the portal venous branches; Grade 3 fatty liver in which the diaphragmatic outline is obscured.

j DM was defined by a history of DM or taking anti-hyperglycemic drugs (oral agents, insulin) or an FBS≥126 mg/dL, without any history of DM type I.

### Cohort attrition

The 7-year migration rate was low; only 46 participants (0.7%) emigrated from the cohort catchment area. The attrition of the cohort included another 359 participants who withdrew from the study (**Figure 3**). A 7-year follow-up success rate of 93.0% was achieved with an active follow-up of 5,394 participants.

### Prevalence of diseases

Some basic findings of the enrollment and the first RM are reported in **Table 2**. In both phases, the prevalence of overweight or obesity was higher among women compared to men. In the enrollment phase (2010), 63.9% of men and 80.9% of women were overweight or obese (BMI ≥ 25 kg/m^2^) (χ2 = 391.45, P<0.001). In the first RM (2017), 64.0% of men and 81.2% of women were overweight or obese (χ2 = 373.16, P<0.001).

The most prevalent obesity-related chronic disease in the study was metabolic syndrome (MetS). The prevalence [95% confidence interval] of MetS based on the joint interim statement’ (JIS’) definition was 31.9% [30.1-33.7%] in men and 46.8% [44.6-49.0%] in women in the baseline (χ2 = 105.79, P<0.001), and 33.1% [31.4-46.8%] in men and 35.9% [33.9-37.9%] in women in the first RM (χ2 = 4.19, P=0.041).

The baseline prevalence [95% confidence interval] of NAFLD grades 2 and 3 was 9.1% [7.9–10.2%] and 1.5% [1.0–2.0%] in men, respectively, and 11.1% [9.7–12.5%], and 2.0% [1.4–2.6%] in women, respectively (χ2 = 4.40, P=0.037). The prevalence respectively increased to 16.2% [14.7–17.7%], 5.2% [4.3–6.1%], 15.6% [14.0–17.2%], and 3.0% [2.0–4.0%] in the first RM (χ2 = 23.6, P<0.001). The baseline prevalence of T2DM was 9.1% [8.0–10.1%] in men and 16.4% [14.8–17.9%] in women. After seven years, the prevalence respectively changed to 11.2% [9.5–12.7%] and 20.9% [18.7–23.0%].

### Causes of death

As reported in **Table 3**, 160 participants, with a mean ( ± standard deviation) age of 68.77 ( ± 14.95) years, died during seven years of follow-up, mostly due to CVDs (except cerebrovascular accidents (CVA), 45 cases, 28.1%), cancers (29 cases, 18.1%), CVA (24 cases, 15.0%), and motor vehicle accidents (10 cases, 6.3%). Also, 52 cases (32.5%) died due to other causes of death (including unknown causes).

**Table 3:** Count and causes of death in the Amol Cohort Study follow-up phase, 2011–2017.

	Cause of Death ^a^					
Follow-up Year	CVD ^b^	CVA	Cancers	MVA	Other ^c^	Total ^d^
First (2011)	4 (25.0)	2 (12.5)	3 (18.7)	2 (12.5)	5 (31.2)	16 (10.0)
Second (2012)	5 (35.7)	2 (14.2)	2 (14.2)	1 (7.1)	4 (28.5)	14 (8.7)
Third (2013)	5 (21.7)	1 (4.3)	5 (21.7)	1 (4.3)	11 (47.8)	23 (14.4)
Fourth (2014)	10 (28.5)	9 (25.7)	6 (17.1)	1 (2.8)	9 (25.7)	35 (21.9)
Fifth (2015)	4 (20.0)	1 (5.0)	6 (30.0)	2 (10.0)	7 (35.0)	20 (12.5)
Sixth (2016)	15 (38.4)	7 (17.9)	4 (10.2)	1 (2.5)	12 (30.7)	39 (24.4)
Seventh (2017)	2 (15.3)	2 (15.3)	3 (23.0)	2 (15.3)	4 (30.7)	13 (8.1)
Total	45 (28.1)	24 (15.0)	29 (18.1)	10 (6.3)	52 (32.5)	160 (100.0)

CVA, cerebrovascular accident; CVD, cardiovascular disease; MVA, motor vehicle accident.

a Numbers represent “count (percentage in the row)”.

b Excluding cerebrovascular accidents.

c Including unknown causes of death.

d Numbers represent “count (percentage in the column)”.

## Discussion

The primary phase of the AmolCS was conducted in 2009 to evaluate obesity-related metabolic disorders and CVDs. The prevalence of obesity and overweight was relatively high in the first RM (64.0% in men and 81.2% in women). The prevalence of T2DM raised after seven years, particularly in women. According to our observation, about 20.9% of northern Iranian women had T2DM in 2017. The prevalence of NAFLD (grades 2 and 3) also increased after seven years. In 2017, moderate or severe NAFLD was prevalent in about 16.2% and 5.2% of northern Iranian men, respectively. NAFLD prevalence in the present study is comparable to the prevalence in Asian countries ranging from 15% to 40% (39). Analyses of data from the PERSIAN cohort study reported a nationwide prevalence of diabetes among Iranian adults about 15% during 2014-2020 (40). The prevalence of T2DM has grown in Iran as well as other parts of the Middle East (41) which is mostly attributed to the increased prevalence of obesity among adults in the last decades (42).

Most participants (61.2%) died from cardiovascular events (CVD and CVA) and cancers. We cooperated with the city municipality to present these findings to the public. The findings call for policy-making and attending to these diseases in the northern Iranian population with simple, cost-effective preventative measures and appropriate diagnostic and therapeutic procedures.

Other studies are also nested in and published based on the cohort. We investigated the prevalence of MetS, T2DM, and NAFLD in our sample population (9, 12, 43). We also obtained the optimal cutoff points for the homeostatic model assessment of insulin resistance (HOMA-IR) and various obesity measures in diagnosing T2DM, MetS, and NAFLD (44–53). We compared various CVD risk assessment tools and evaluated the associations between NAFLD, MetS, and 10-year CVD risk as estimated by well-known international risk assessment tools (45, 54–56). Additionally, we investigated the prevalence and risk factors of hepatitis C in northern Iran (57). We aim to develop and tailor the appropriate CVD risk assessment tools and obesity measures for northern Iranians in the future.

Some aspects of the study design are novel. For instance, we have focused on NAFLD and other obesity-related metabolic disorders in this cohort. In addition, aiming to develop and validate LMIC-tailored risk assessment tools, we covered a unique population not represented in existing CVD cohorts. We also adopted a multidisciplinary approach across levels of ministerial hierarchy to conduct the study and reduce cohort attrition.

The initial comprehensive assessment and subsequent follow-up of 5,394 participants in seven years with a multidisciplinary approach was the main strength of the AmolCS. The study involved data collection with frequent updates over seven years with a relatively high participant retention rate. There are several socioeconomic and cultural barriers impeding the coherence of participants in a longitudinal study (58). In the AmolCS, we cooperated with the district health network to overcome these potential barriers. The health network universally covers the population regardless of socioeconomic status. Therefore, financial problems were not important barriers to participation. Since all the personnel of the health network and the research center were trained residents, we faced no cultural and communicational challenges (e.g., language). In addition to in-person visits, another factor supposed to yield a high retention rate in the cohort was a highly literate and well-educated population eagerly taking part in health surveillance. The sample population was also relatively stable, with minimal migration.

Physical activity, tobacco smoking and other personal habits, diet, quality of life, stressful life events, biochemical profiles, liver ultrasonography, and ECG were evaluated in the enrollment and/or RM phases. We also cooperated with several specialists and sub-specialists to assess the participants, including those showing abnormalities in clinical and para-clinical evaluations. Obtained data encompassed demographic variables, medical conditions, lifestyle behaviors, and risk factors for the diseases of interest. The study outcomes offer evidence for further studies and inform practice and policy regarding NCDs in Iran.

As with any cohort study, there are a few limitations related to the present study. The lack of data on electrocardiography, blood counts, glycated hemoglobin (HbA1C), C-reactive protein (CRP), thyroid-stimulating hormone (TSH), triiodothyronine (T3), thyroxine (T4), blood urea nitrogen (BUN), creatinine, calcium, phosphorus, and 25-hydroxycholecalciferol (vitamin D) in the enrollment, and viral hepatitis markers in the first RM was the main drawback of the study, making the exclusion of prevalent diseases and the estimation of incident diseases difficult. For example, we could not use HbA1C to estimate T2DM incidence before 2017, but it would be feasible to estimate the incidence from 2017 to 2023 when the data is available. Another limitation is the use of ultrasound to assess liver fat content. Although liver biopsy is a gold standard for diagnosing NAFLD, we use sonography for evaluating NAFLD due to the risks associated with liver biopsy and the impossibility of applying it in population-based studies. Ultrasound is a non-invasive and widely available imaging technique and is highly accurate for patients with moderate to severe steatosis (59).

Recall bias and false reports are other potential sources of limitation in the study. We used approaches such as self-reporting and robust reviewing of medical documents to reduce information bias.

## Conclusion

The current study has focused on obesity-related metabolic disorders in a general population not represented in existing cohorts. A multidisciplinary approach for enrolling and following up helped to obviate barriers to participation. The most prevalent obesity-related chronic disease in the study was metabolic syndrome. The findings of AmolCS highlight significant disparities in various health parameters between men and women. Across both phases, women exhibited a higher prevalence of obesity, metabolic syndrome, and T2DM. These differences underscore the importance of considering gender-specific factors in health assessment and intervention strategies. The findings of AmolCS call for policy-making and developing appropriate preventive and therapeutic measures and offer an evidence base for future studies to reduce the burden of non-communicable diseases in northern Iran.

## Data availability statement

The raw data supporting the conclusions of this article will be made available by the authors, without undue reservation.

## Ethics statement

The studies involving humans were approved by Iran University of Medical Sciences (reference number: IR.IUMS.REC.1397.162). The studies were conducted in accordance with the local legislation and institutional requirements. Written informed consent for participation in this study was provided by the participants’ legal guardians/next of kin.

## Author contributions

NM: Writing – review & editing, Writing – original draft, Supervision, Resources, Formal analysis, Data curation, Conceptualization. FZ: Writing – review & editing, Validation, Supervision, Resources, Project administration, Data curation, and Conceptualization. MM: Writing – review & editing, Resources, Methodology, Investigation. HA: Validation, Writing – review & editing, Resources, Methodology. FR: Writing – review & editing, Resources, Methodology. HK: Investigation, Writing – review & editing, Resources. HP: Methodology, Writing – review & editing. RS: Validation, Writing – review & editing, Methodology. GHA: Writing – review & editing, Methodology. DP: Writing – review & editing, Methodology. BR: Investigation, Writing – review & editing. MML: Writing – review & editing, Investigation. MK: Validation, Writing – review & editing, Investigation. ZK: Writing – review & editing, Investigation. MRS: Writing – review & editing, Investigation. AD: Writing – original draft, Resources, Methodology, Writing – review & editing, Investigation. BA: Writing – review & editing, Investigation. FST: Writing – review & editing, Investigation. EG: Writing – review & editing, Investigation. SHH: Writing – review & editing, Investigation. MHK: Writing – review & editing, Investigation.
